# Determine the factors affecting the time to recovery of children with bacterial meningitis at Jigjiga university referral hospital in the Somali Regional State of Ethiopia: using the parametric shared frailty and AFT models

**DOI:** 10.1186/s13104-024-06740-9

**Published:** 2024-03-19

**Authors:** Daud Hussein Adawe, Dagne Tesfaye Mengistie

**Affiliations:** Department of Statistics, College of Natural and Computational Science, Jigjiga University, Jigjiga, Ethiopia

**Keywords:** Bacterial meningitis, Survival data, Parametric frailty model, AFT Model, Time to recovery

## Abstract

**Background:**

Neisseria meningitides, Streptococcus pneumonia, and hemophilic influenza type B are frequently linked to bacterial meningitis (BM) in children. It’s an infectious sickness that kills and severely mobilizes children. For a variety of reasons, bacterial meningitis remains a global public health concern; most cases and deaths are found in Sub-Saharan Africa, particularly in Ethiopia. Even though vaccination has made BM more preventable, children worldwide are still severely harmed by this serious illness. Age, sex, and co-morbidity are among the risk variables for BM that have been found. Therefore, the main objective of this study was to identify the variables influencing the time to recovery for children with bacterial meningitis at Jigjiga University referral hospital in the Somali regional state of Ethiopia.

**Method:**

A retrospective cohort of 535 children with bacterial meningitis who received antibiotic treatment was the subject of this study. Parametric Shared Frailty ty and the AFT model were employed with log likelihood, BIC, and AIC methods of model selection. The frailty models all employed the patients' kebele as a clustering factor.

**Results:**

The number of cases of BM declined in young children during the duration of the 2 year, 11 month study period, but not in the elderly. Streptococcus pneumonia (50%), hemophilic influenza (30.5%), and Neisseria meningitides (15%) were the most frequent causes of BM. The time to recovery of patients from bacteria was significantly influenced by the covariates male patients (ϕ = 0.927; 95% CI (0.866, 0.984); p-value = 0.014), patients without a vaccination history (ϕ = 0.898; 95% CI (0.834, 0.965); P value = 0.0037), and patients who were not breastfeeding (ϕ = 0.616; 95% CI (0.404, 0.039); P-value = 0.024). The recovery times for male, non-breastfed children with bacterial patients are 7.9 and 48.4% shorter, respectively. In contrast to children with comorbidity, the recovery time for children without comorbidity increased by 8.7%.

**Conclusion:**

Age group, sex, vaccination status, co-morbidity, breastfeeding, and medication regimen were the main determinant factors for the time to recovery of patients with bacterial meningitis. Patients with co-morbidities require the doctor at Jigjiga University Referral Hospital to pay close attention to them.

## Introduction

### Background of the study

A serious infection of the central nervous system known as bacterial meningitis (BM) can have either a short- or long-term outcome [1, 2]. Clinically speaking, meningitis is an inflammation of the meninges that surround the brain and spinal cord [3]. This inflammation can be caused by a variety of agents, including viruses, fungi, protozoa, bacteria, and encapsulated bacteria. The most well-known and frequent of these are *Streptococcus pneumonia, Hemophilic influenza, and Neisseria meningitides* [4, 5]. Meningitis causes significant neurosensory consequences and a high fatality rate (about 117,000 deaths annually globally). The disease’s median incidence in children under five is 34 cases per 100,000 children annually [6]. The incidence varies by global region; in the Americas, it is 16.6 per 100,000 children year, whereas in Africa, it is 143.6 per 100,000 children annually. With a range of 31.3% in the Africa region and 3.7% in Southeast Asia, the median case fatality rate is 14.4% [2, 7].

The symptoms of fever, altered mental status, headache, and nuchal rigidity are suggestive of bacterial meningitis, though many people with the illness do not exhibit any of these symptom [8]. A spinal tap is necessary to get cerebrospinal fluid in order to make a conclusive diagnosis of meningitis [1, 5]. Meningitis patients frequently have low blood sugar, elevated white blood cell counts, and elevated protein levels in their fluid. It might be possible to determine which bacteria caused the meningitis by analyzing the fluid. By cultivating the CSF sample, bacterial meningitis is diagnosed. After measuring the opening pressure, the fluid should be submitted for microbiology (i.e., Gram stain and cultures), chemistry (i.e., CSF glucose and protein), and cell count (and differential count) [1, 6]. The traditional trio of meningitis symptoms fever, stiff neck, and altered mental status occurs in just 41% of cases of bacterial meningitis. The majority of patients who experience the triad are older. At least one of these symptoms will be present in 70% of individuals [6]. A method carried out in sterile conditions with the goal of preventing the entry of undesired organisms or bacterial pollutants into an environment is known as an aseptic technique. To prevent contamination of lab workers, cultures, and equipment, this is a crucial microbiological lab procedure [6, 9]. In the microbiological laboratory, both chemical and physical sterilization techniques are used to guarantee that the materials and equipment are free of germs. Sanitization accomplishes this by lowering contamination to acceptable levels through the use of any cleaning method that mechanically eliminates germs and other debris. While antiseptics are used to disinfect flesh, disinfectants are administered to inanimate surfaces, medical equipment, and other man-made objects [1, 9].

Meningitis has been one of the most dreaded infectious diseases throughout the nineteenth and twentieth centuries, and it is now a top priority for public health [4, 6]. At least 1.2 million instances of invasive illness are thought to occur annually around the world, and invasive meningococcal disease (IMD) is thought to be responsible for 135,000 of those deaths [10]. The public health system is severely taxed by the disease burden in nations with high endemicity [11]. In low-income nations, where the prevalence of bacterial meningitis is highest, there is a higher risk of long-term debilitating sequelae, such as cognitive impairment, bilateral hearing loss, motor impairment, seizures, visual impairment, hydrocephalus, and amputation of limbs owing to tissue necrosis [1, 6, 12].

*Streptococcus pneumonia, Hemophilic influenza,* and *Neisseria meningitides* are the primary etiological agents of bacterial meningitis outside of the neonatal period, and it continues to be a significant source of morbidity and mortality [2, 11]. These agents are extremely significant in sub-Saharan Africa's meningitis belt, where epidemics of the disease happen every 8–12 years. For instance, there were 42 cases in the Burkina Faso outbreak of 1996, with a 10% case fatality rate (CFR) [13]. In Ghana, there were 18551 cases in 1997, and 8% of them resulted in death [12]. The Meningococcal Meningitis Case Fatality Rate (CFR) is estimated by the World Health Organization (WHO) to be 10%, or 500,000 cases, each year, with 27,000 of those cases occurring in African regions [14]. Bacterial meningitis (BM) alone accounts for roughly 6–8% of all hospital admissions in Ethiopia, and its case fatality rates can reach 22–28%. For the past many decades, Ethiopia’s health has continued to be a major concern [15, 16]. Studies on those who have recovered from bacterial meningitis have revealed a wide spectrum of neurological, cognitive, and behavioral consequences [17].

Systemic infections are predisposed to by malignant illnesses. Important factors include malnourishment, immunosuppressive therapy, and persistent venous catheters. Patients with leukemia, lymphomas, and those who have undergone neurosurgical procedures for brain tumors are more susceptible to meningitis [5, 8]. In this demographic group, *Streptococcus pneumonia, hemophilic influenza, and Neisseria meningitides* are the most prevalent pathogens. An intracranial space-occupying process, thrombocytopenia, or an unusual clinical presentation might occasionally cause a delay in the proper diagnosis and appropriate management [18].

Recipients of transplant organs are more susceptible to invasive pneumococcal infections, which can cause sepsis and meningitis. Prior to transplantation, pneumococcal vaccination lowers the risk. *L. monocytogenes and Nocardia spp.* are other culprits that can cause meningitis in this population, particularly in cases where there are several brain abscesses [19]. Hypo-or asplenia, characterized by splenic dysfunction or absence, puts a person at risk for invasive infections from encapsulated bacteria, including *S. pneumonia and H. influenza*. Splenectomy is one way to treat acquired hyposplenism. HIV infection, sickle cell anemia, graft-versus-host disease, allogenic bone marrow transplantation, and celiac illness can also cause it to function [20]. Just 2.5% of community-acquired meningitis cases had asplenia, which is linked to a high death rate of 25% and persistent neurologic sequelae of 58% [18].

The time to completely recover children from bacterial meningitis (BM) from the day of diagnosis was the event of interest in this study [21]. The current study identified factors influencing children with bacterial meningitis (BM) recovery time using parametric shared frailty models and an AFT parametric model. The shared frailty model, a variant of the Cox PH model called the frailty model, takes into account any extra heterogeneity in the data [22].

## Data and material

### Description of the study area

The study was carried out at Jigjiga University Referral Hospital, which is located 635 km from Addis Ababa, the capital city of Ethiopia, in the eastern section of the country, in the regional Jigjiga town of the Somali regional state [23]. The children diagnosed with bacterial meningitis comprise the study population under inquiry.

### Study design and population

A retrospective cohort research was conducted at Jigjiga University Referral Hospital in the regional state of Somali. The study's target population consisted of the children less 18 year being treated at Jigjiga University Referral Hospital in Somali Regional State who had bacterial meningitis. The study was conducted between 10th of August 2023 and 30th of August, 2023 among children who were admitted to the hospital with sever bacterial meningitis from September 1, 2019 to July 20, 2023 at Jigjiga University referral hospital in Somali regional state. All children patients who were admitted for this referral hospital due to case of bacterial meningitis during the above stated dates was included the study. There were 535 children bacterial meningitis patients enrolled in this study.

### Source of data

The secondary data used in this study was collected from Jigjiga University Referral Hospital in the Somali Regional State of Ethiopia for children less 18 year who had bacterial meningitis (BM).

### Inclusion and exclusion criteria

#### Inclusion criteria

All instances of bacterial meningitis in children who had a clinical diagnosis and started treatment were included in the study.

#### Exclusion criteria

The study did not include patients who were admitted but whose bacterial meningitis was not clinically confirmed. After the initial diagnosis, children also developed fungal, viral, or other forms of meningitis.

#### Data extraction and measurement

Baseline data was being extracted from the department of clinical pediatric registration book on which laboratory findings after investigation and recorded existing patients recorded card in the hospital. The whole patients who admit and diagnosed at Jigjiga university referral hospital. Baseline parameters that was extracted were time, age, sex, drug regimen (antibiotics), pathogen (organism caused the disease), residence place, breastfeeding, co-morbidity, vaccination status and kebele. The patients data about whether cured or not were extracted from daily follow up chart and the time to cure was calculated by subtracted the date of diagnosed from the date of discharge the hospital.

### Study variables

#### Response variable

The outcome or dependent variable taken into account in this study for children with bacterial meningitis was the length of hospitalization from the first day of the patient's diagnosis at the hospital to the last day of discharge from the hospital.

$$ {\text{Status~~~}}\left\{ \begin{gathered}   0\,\,if~event~\left( {occure~when~bacterial~meningitie~childeren~patient~recovery~during~followup} \right) \hfill \\   1\,\,if~censored\left( {died,{\text{~transferred~to~other~hospitals}},{\text{~lost~and~dropped~before~death}}} \right) \hfill \\  \end{gathered}  \right. $$

#### Independent or explanatory variables

The independent factors included in this study were age, sex, immunization status, nursing practices, organisms or infections, co-morbidity, medication regimen, and place of residence.

#### Method of data analysis

Several methods were employed in this study to examine the factors affecting the time to recovery of children with bacterial meningitis at Jigjiga University Referral Hospital in the Somali Regional State of Ethiopia by Using the parametric shared frailty and AFT models. Among those for comparing the survival experiences of two or more groups, a nonparametric test was provided. Kaplan–Meier survival function was non-parametric estimation, survival distribution estimation from a sample, and survival distribution were a few of these descriptive statistics. The most common and widely used test was the log-rank test. The Cox proportional hazards model was semi-parametric methods used in multivariable analysis. To further better address the goal of the investigation, AFT and parametric shared frailty models with loglikelhood, the Akaike Information Criterion (AIC), and the Bayesian Information Criterion (BIC) techniques of model selection were applied [1, 24]. R and STATA (version 17) were the programs used during this study to analyze statistical data.

## Results

A total of 535 children with microbiologically confirmed bacterial meningitis were included in this study. Out of the 535 children with bacterial meningitis, 86 (16.6%) had their cases censored since it was unknown how many of them actually survived; the remaining 449 (83.4%) cases had known outcomes such as recovery from bacteria. Among 221 (41.3%) female children of bacterial patients, 35 (39.3%) of the female patients died, were transferred to other hospitals, lost, or dropped before death, which means they censored or did not recover from bacterial disease, whereas 186 (41.7%) of the female children of bacterial patients recovered from disease or lived. From a total of 314 (58.7%) male patients, 54 (60.7%) male bacterial patients were not recovering from the disease, while 260 (58.3%) male bacterial patients were recovering from the disease. In addition to this, the median recovery times for females and males were 14 and 12 days, respectively.

Out of 158 (29.5%) children with no vaccination status, 61 (68.5%) were censored, but 97 (21.7%) recovered from the disease while Among the 377 (70.5%) of children of bacterial patients that have vaccination status, 28 (31.5%) of children were censored, and 349 (78.3%) of the children of patients recovered from bacterial disease during follow-up. Among a total of 267 (49.9%) children bacterial patients who have comorbidities, 19 (21.3%) were censored, and the rest, 248 (55.6%) of the children with comorbidities, recovered from disease. Patients with co-morbidities spent an average of 13 days in the hospital, of which 73.88% recovered from disease; in contrast, patients without co-morbidities remained an average of 12 days, of which 92.88% recovered.

Among 505 (94.4%) of children who live in urban areas, 82 (92.1%) of the bacterial patients were censored, but 423 (94.8%) recovered from disease. While, out of 30 bacterial patients who lived in rural areas, 7 (7.9%) of the children were censored, and the rest, 23 (5.2%), were removed from bacterial meningitis. In comparison to rural patients, who had a median recovery time of 15 days and a recovery rate of 76.67%, urban patients had a median recovery time of 12 days. *Streptococcus pneumonia (*50*%), hemophilic influenza (30.5%), and Neisseria meningitides (15%)* were the most frequent causes of *BM. Streptococcus pneumonia and hemophilic influenza* were the pathogens or organisms that caused the disease in the patients, and both had a median time to recovery of 12 days, with respective recovery rates of 85.19 and 78.53%. Another pathogen with a median recovery time of 13 days and an overall recovery rate of 83.75% is Neisseria meningitides.

### Non‑parametric survival analysis

**Fig. 1:**
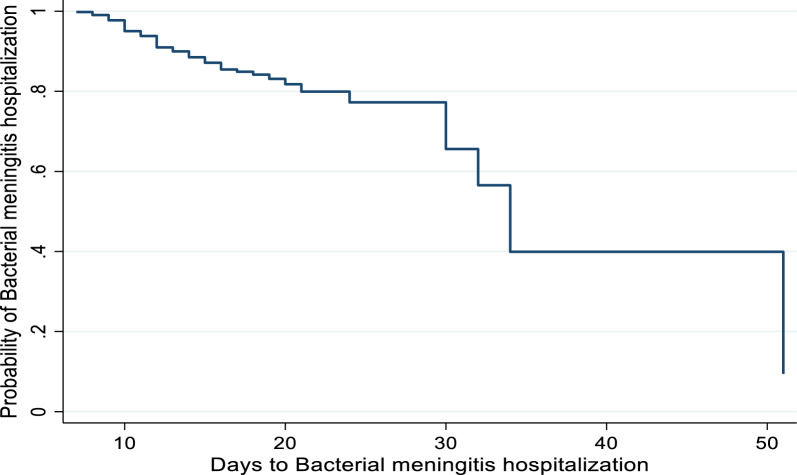
Survivorship of Bacterial meningitis hospitalization

**Fig. 2:**
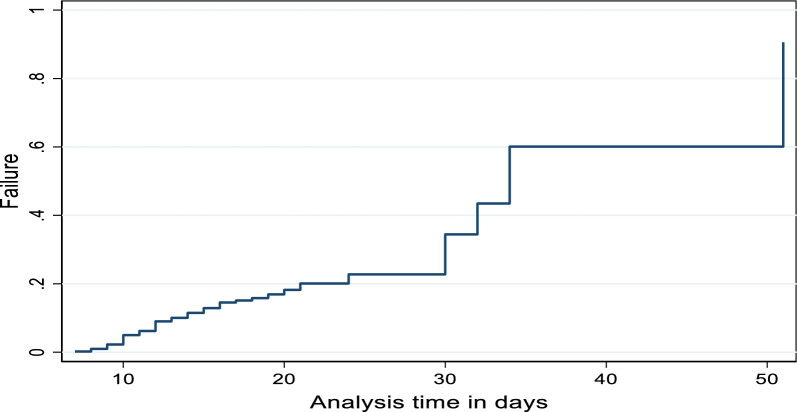
Survival failure function plot for the time to survive of BM Patient

**Fig. 3:**
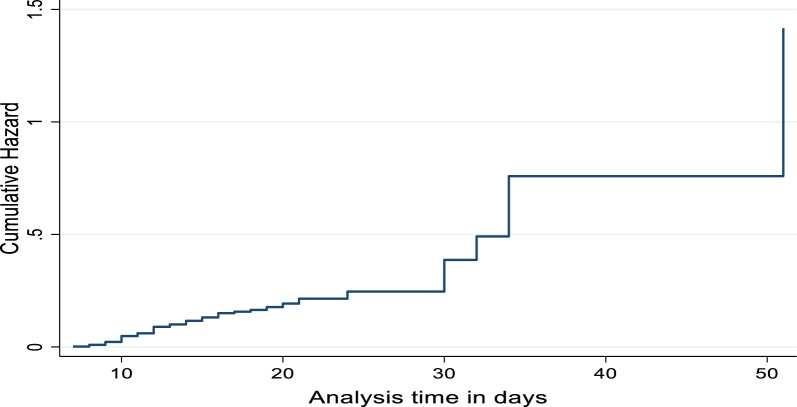
cumulative Hazard plot for the time to survive of BM Patient

Plots of the Kaplan-Meir curves for the survival, survival failure, and cumulative hazards experienced for the time to survive of the bacterial patient were used for the non-parametric analysis, as illustrated in Figs. 1, 2, 3, respectively. The outcome showed that the survival plot initially declined at an increasing rate and then occasionally reduced much more. This suggests that the majority of bacterial meningitis patients will get treatment shortly. On the other hand, the hazard plot started out growing faster and kept getting bigger with time.

### Survival comparison of different groups of BM patients

**Fig. 4:**
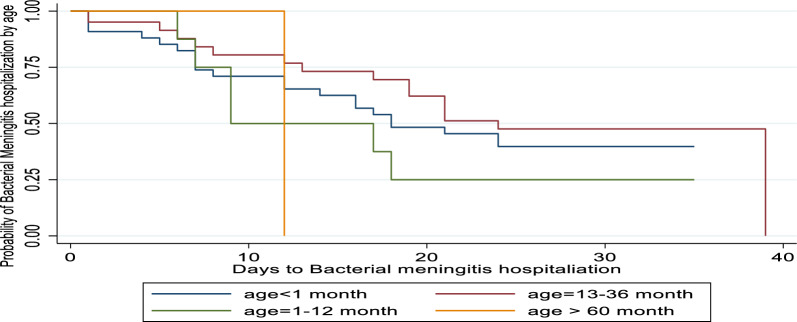
survival function of age bacterial patient

The age group older than 60 months has a higher survival rate than the age group younger than 1 month, especially in the intermediate stages when everything is essentially the same at the beginning and the end. This suggests that patients older than 60 months have a higher chance of recovering more slowly at a given point in time than patients younger than one month. Table 1 above shows that the group older than 60 months had a substantially different effect from the group younger than 1 month, whereas the other patient age groups did not exhibit a significantly different survival function at 5% level of significance see Fig. 4.

**Table 1:** Baseline characteristics of covariates and median duration for survival time of bacterial patient

Variable	Category	No of patients	Median	95% CI	Status of the patients			
					Censored		Even (Recovery)	
					Count	Percent (%)	Count	Percent (%)
Sex	Female	221	14	[13, 15]	35	39.3	186	41.7
	Male	314	12	[11, 13]	54	60.7	260	58.3
Age group	< 1 month	3	8	[7, 9]	2	2.2	2	0.4
	1–12 months	243	12	[12, 13]	35	39.3	209	46.9
	13–36 months	109	12	[11, 13]	19	21.3	90	20.2
	37–60 months	48	12	[11, 14]	11	12.4	37	8.3
	> 60 months	132	12	[13, 17]	7	7.9	41	9.2
Vaccination status	No	158	13	[12, 14]	61	68.5	97	21.7
	Yes	377	12	[11, 13]	28	31.5	349	78.3
Pathogens	Streptococcus pneumonia	270	12	[11, 14]	22	24.7	166	37.2
	Hemophilic influenza	163	12	[11, 14]	58	65.2	202	45.3
	Neisseria meningitides	80	13	[12, 14]	9	10.1	64	14.3
	Others	22	15	[12, 16]	0	0.0	14	3.1
Drug regime	Ceftriaxone and vancomycin	120	14	[12, 15]	24	27.0	96	21.5
	Ceftrazidime and vancomycin	44	13	[11, 18]	10	11.2	34	7.6
	Ceftriaxone and dexamethine	234	12	[12, 14]	36	40.4	198	44.4
	Cefoperatone and sulbactan	24	13	[11, 14]	2	2.2	12	2.7
	Ciprofloxacin and ceftriaxone	2	12	[10, 13]	1	1.1	1	0.2
	Ampiciline and gentamycin	110	12	[11, 13]	14	15.7	96	21.5
	Others	11	15	[11, 17]	2	2.2	9	2.0
Residence place of the patients	Rural	30	15	[13, 18]	7	7.9	23	5.2
	Urban	505	12	[12, 13]	82	92.1	423	94.8
Comorbidies	Yes	267	13	[11, 13]	19	21.3	248	55.6
	No	268	12	[12, 14]	70	78.7	198	44.4
Breast feeding of the patients	Yes	180	12	[13, 15]	52	58.4	142	31.8
	No	355	13	[11, 13]	37	41.6	304	68.2

*CI* Confidence interval

**Fig. 5:**
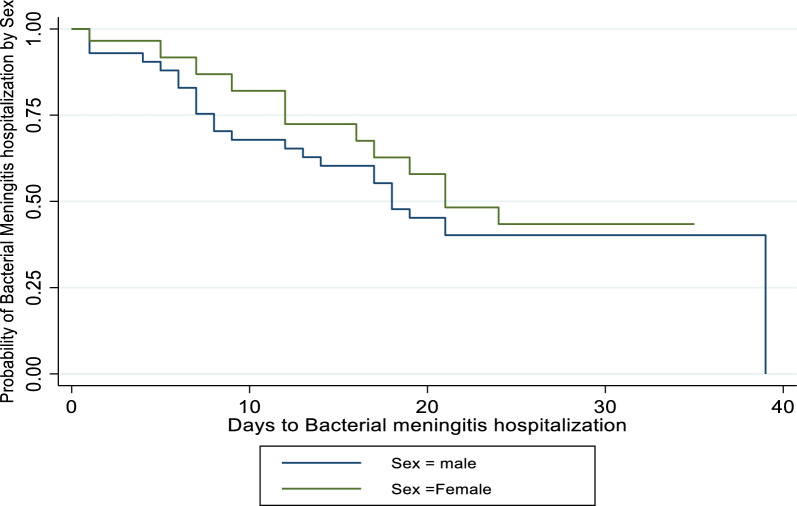
survival function of sex of bacterial patient

Male patients have a lower survival rate than female patients, especially at midterm. However, they are all more similar at the beginning and at the bending times depicted in Fig. 5. This suggests that male patients have a lower probability of recovering at a given time than female patients do.

**Fig. 6:**
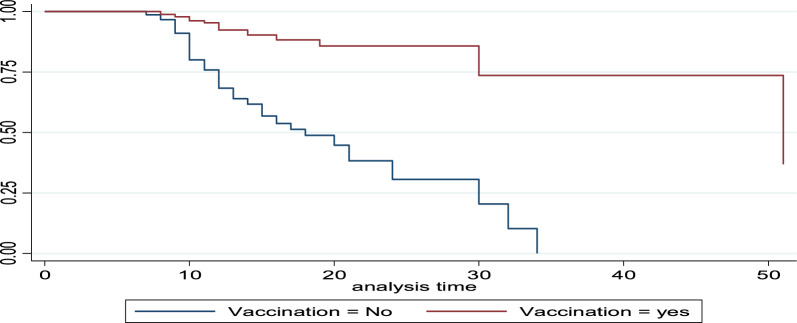
survival function of vaccination by time to recovery bacterial patient

The survival rate for the vaccinated group of patients is lower than that of the non-vaccinated group, especially in the midterms, but everything is the same at the beginning and at the end (see Fig. 6). This shows that the probability of the time to recovery for the vaccinated group is lower than when we compare it to the non-vaccinated group, as the probability of curing time for the non-vaccinated group is higher than that of the vaccinated group.

### Cox proportional hazard regression model

All covariate Cox proportional hazards models were fitted. The univariate analysis revealed that comorbidities, sex, age, immunization status, breastfeeding status, and comorbidity were all statistically significant factors for the variable of interest. Regarding the other criteria, such as residence and trends in drug use, there was no statistically significant difference.

### Model diagnosis for Cox proportional hazards model

Table 2 illustrates how the log-linearity and proportional hazards assumptions of the Cox regression model were examined in the current investigation. The log-linearity test demonstrated that the log hazard, also known as the log cumulative hazard, and the covariate had a linear relationship. The proportional hazard test carried out in this work indicates that the ratio of the hazard function for two persons with different regression covariates does not change over time. The Wald chi-square test statistic was significant, as shown by the global fit test in Table 2, which disproves the proportional hazards assumption.

**Table 2:** Result of the test of proportionality assumptions for each covariate in the final model

Variable	Rho	Std. Err.	Z	P > z	[95% Conf	Interval]
Sex	1.193867	0.1157498	1.83	0.068	0.9872541	1.443721
Age group	0.9935444	0.0323247	− 0.20	0.842	0.9321668	1.058963
Vaccination	1.468227	0.175882	3.21	0.001	1.160984	1.85678
Pathogens	0.9090509	0.0553318	− 1.57	0.117	0.8068216	1.024233
Drug regime	1.038888	0.02757	1.44	0.151	0.9862326	1.094354
Residence	1.009224	0.2262703	0.04	0.967	0.6503487	1.566134
Breastfeed	1.363629	0.1638869	2.58	0.010	1.077445	1.725828

Global test chi2 = 11.39, df = 19, p-value = 0.9100

### Model diagnosis with graphical method

**Fig. 7:**
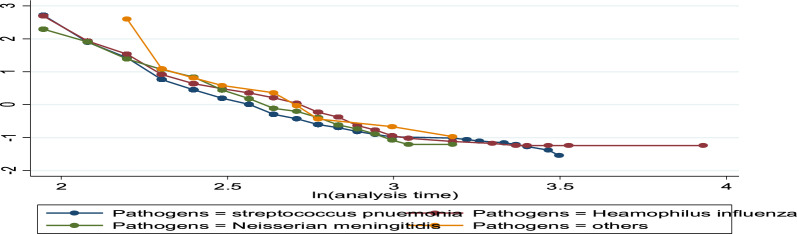
log–log plot of survival of pathogen caused disease of time to recovery of bacterial

**Fig. 8:**
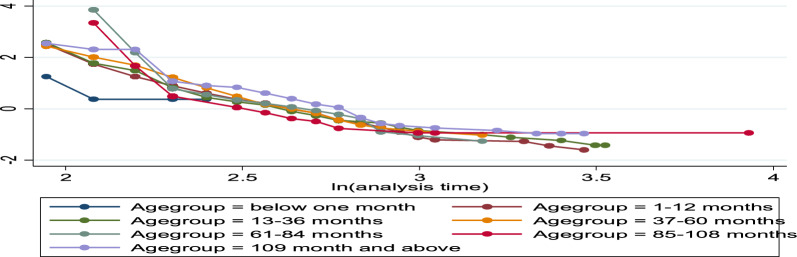
log–log plot of survival of age group patient of time to recovery of bacterial

**Fig. 9:**
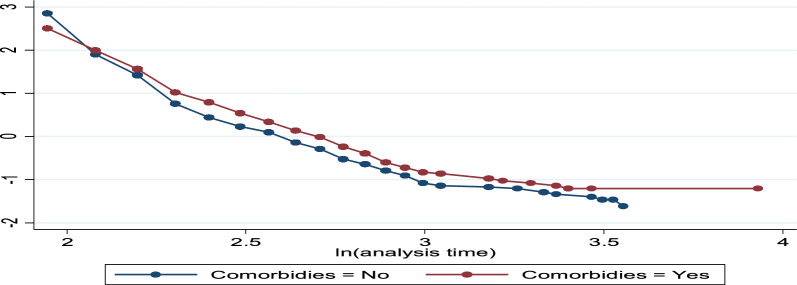
log–log plot of survival of comorbidity patient of time to recovery of bacterial

**Fig. 10:**
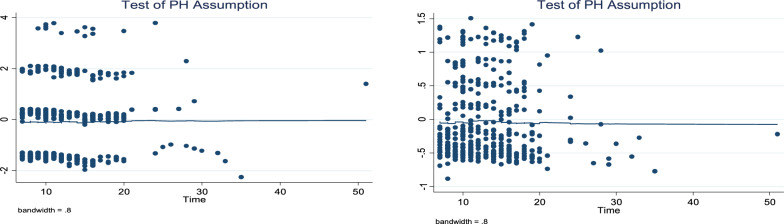
Plot of the scaled schoenfeld for pathogen cause and age group of time to recovery of bacteria

The log (time to recovery) vs. log (-log(s (t))) plots were not parallel to one another. As a result, the proportionate hazards assumption is broken (see Figs. 7, 8, 9). The smoothed plot, which resembles a straight line with some departure from the horizontal line, indicates that the residuals follow a systematic pattern and are not random, as demonstrated by the plot of the scaled Schoenfeld. Consequently, Fig. 10’s proportionate hazards assumption is violated.

### Accelerated failure time model results

#### Unavailable analysis

The covariates of breastfeeding, co-morbidity, and vaccination status of the patients are significant in all of the models that were used.

#### Multivariate AFT analysis

Covariates that were no longer significant in the multivariate analysis were eliminated from the model using the backward elimination approach. As a result, the drug regimen and living address were dropped or omitted. The effect of interaction terms was further investigated at the 10% level of significance and was shown to be statistically insignificant in the multivariable lognormal AFT model. The primary effects of the covariates vaccination status, patient breastfeeding, sex, age, and comorbidity were retained in the final model. All of the AFT models are listed in Table 3, along with the corresponding log likelihood, BIC, and AIC values.

**Table 3:** Compares AFT and Frailty models using data on time to recovery status

Distribution	AIC	BIC	log-likelihood
Exponential	29672.82	29779.60	− 11148
Weibull	20063.76	20178.13	− 11256
Log logistic	20063.76	20178.13	− 11256
Generalized gamma	19883.78	20005.78	− 11126
Log-normal	***19865.06***	**19997.29**	**− 11025**

Baseline hazard function	Frailty distribution	AIC	BIC	DIC
Exponential	Gamma	3324.760	418.870	371.710
	Inverse Gaussian	3325.506	389.378	350.024
Weibull	Gamma	2824.988	392.919	353.197
	Inverse Gaussian	2813.650	394.808	354,871
Lognormal	Gamma	2691.597	410.978	379.897
	Inverse Gaussian	**2674.080**	**314.987**	**332.510**
Log logistic	Gamma	2692.105	399.389	379.024
	Inverse Gaussian	2675.287	389.991	396.179

Bold values indicate selected model that means Inverse Gaussian models was the appropriated for our study

*AIC* Akaike’s information criteria, *BIC* Bayesian information criterion, *DIC* deviance information criterion

Table 3 shows that AIC, BIC, and log-likelihood were used to evaluate AFT models like Weibull, exponential, log-logistic, and lognormal distributions to see which one best fit the data. Consequently, the AIC and BIC for the lognormal distribution were the lowest. For this reason, it has been chosen for the current study's univariate and multivariate data analysis.

#### Univarable analysis for frailty models

The covariates co-morbidity, vaccination status, and breastfeeding are significant in all of the univariable analysis models at the 0.05 level of significance. Age group and sex are also significant at 5% for most of the models except exponential baseline with gamma and inverse Gaussian frailty distribution, where drug regime is significant lognormal at baseline with inverse Gaussian frailty distribution. In all models, the pathogen variables and the residence site are not relevant for the recovery time at the 5% level of significance.

In the univariable analysis, significant covariates were identified and used in the multivariate AFT baseline hazard function with the gamma and the inverse Gaussian frailty distributions. The model with the lowest AIC for the bacterial meningitis data set was lognormal at baseline with an inverse Gaussian frailty distribution. The lognormal inverse Gaussian frailty model, as presented in Table 3, had the lowest AIC and BIC values of all the parametric frailty models (AIC = 2674.080, BIC = 314.987). This suggests that the model was comparatively the most effective in explaining the children's bacterial meningitis data set.

In the lognormal inverse Gaussian frailty model, the shape parameter was 2.184. This number is more than one unity, indicating that the hazard function’s shape grew initially before eventually decreasing. According to Table 4, the heterogeneity within clusters is 22.1%, whereas the heterogeneity in the population of clusters estimated for this best model is ϴ = 0.98, which is significant at the 5% level of significance. 13 days was the patients’ overall median recovery period from bacterial meningitis. Table 4 presents the findings of a study using the lognormal inverse Gaussian frailty model. The factors that were shown to be significant at the 5% level of significance were age group, sex, co-morbidity, vaccination status, breastfeeding, and medication regimen.

**Table 4:** Multivariable analysis using lognormal inverse Gaussian frailty model

Variable names	Category	Coefficient	S.E	ϕ	95% CI	P-value
Sex	Female(ref.)			1.00		
	Male	− 0.079	0.033	0.924	[0.866, 0.984]	0.014*
Age	< 1 month					
	1–12 months	0.340	0.213	1.405	[0.926, 2.133]	0.110
	13–36 months	0.323	0.215	1.381	[0.906, 2.105]	0.130
	37–60 months	0.299	0.219	1.349	[0.878, 2.072]	0.170
	> 60 months	0.484	0.215	1.623	[1.065,2.4747]	0.024*
Vaccination status	No vaccinated(ref.)					
	Yes vaccinated	− 0.109	0.037	0.898	[0.834, 0.965]	0.003*
Presence of comorbidity	Yes (ref.)					
	No	0.087	0.033	1.098	[1.022, 1.164]	0.008*
Breast feeding of patients	Yes (ref.)					
	No	0.484	0.215	0.616	(0.404, 0.939)	0.024*
Drug regime	Ceftriaxone plus vancomycin					
	Ceftrazidime plus vancomycin	− 0.045	0.066	0.956	(0.839, 1.089)	0.500
	Ceftriaxone plus dexamethasone	− 0.070	0.042	0.932	(0.850, 1.012)	0.092
	Cefoperatone plus sulbactan	− 0.064	0.104	0.938	(0.765, 1.151)	0.450
	Ciprooxacin plus ceftriaxone	− 0.135	0.296	0.874	(0.489, 1.559)	0.650
	Ampicillin plus gentamycin	− 0.128	0.052	0.880	(0.795, 0.974)	0.013*
	Others	0.060	0.116	1.062	(0.846, 1.332)	0.600
	Constant	2.330	0.215	10.282	(6.742, 15.680)	0.000

p = 0.05 was statistically significant. ϴ = 0.98 ρ = 2.184 τ = 0.221 AIC = 2674.080 *ϕ* Acceleration Factor, *CI* Confidence Interval, *ϴ* Variance of random effect, *τ* Kendall’s Tau S.E standard error, *Ref* Reference. *p* Shape parameter, *AIC* Akaiks Information Criteria

*The significant variable at 5% level

The age group had a significant effect on the time to recovery for bacterial meningitis patients. Hence, comparing the age group of more than 60 months with those of less than 1 month, the expected time to recovery for more than 60-month-old bacterial patients was increased by 12.8% as compared to less than 1 month-old patients, keeping the other covariates constant (ϕ = 1.623; 95% CI (1.065, 2.4747); *p*-value = 0.024). This result indicates that more than 60-month-old children with bacterial meningitis had a prolonged time to recovery as compared to less than a month-old child with bacterial meningitis. Although bacterial meningitis can strike anyone at any age, it most commonly strikes infants, young children, and adolescents. Meningitis and septicemia are more common in particular age groups. Because their immune systems are still developing, young children are more vulnerable than older age groups. Vaccines offer this susceptible age group essential protection and enable young children to safely identify dangerous microorganisms.

Vaccination was a significant factor in the time to recovery of children with bacterial meningitis. Comparing vaccinated children patients with not vaccinated, the expected time to recovery for vaccinated children patients was decreased by 10.9% as compared to not vaccinated children patients, keeping the other covariates constant (ϕ = 0.898; 95% CI 0.834, 0.965); *p*-value < 0.003). This suggests that female children have a better chance of recovering from bacterial meningitis than male children. Because the vaccine does not protect against all strains of the bacteria that can cause the disease. While the vaccine provides very good protection against four of the five most common strains that cause meningococcal disease, it does not provide in all protection from these four strains. Getting the meningococcal vaccine after you've been exposed will not protect you against getting sick from that particular exposure. However, getting the vaccine to protect you against future exposures is a very good idea.

Sex was a significant factor in the time to recovery of children with bacterial meningitis. Comparing male children patients with females, the expected time to recovery for male children patients was decreased by 7.9% as compared to female children bacteria patients, keeping the other covariates constant (ϕ = 0.924; 95% CI 0.866, 0.984); *p*-value < 0.014). This suggests that female children have a better chance of recovering from bacterial meningitis than male children. Because male patients had higher comorbidities and immunosuppressive diseases than female patients, they were also underinsured. There are notable differences between male and female community-acquired meningitis in terms of comorbidities, presentation symptoms and signs, aberrant laboratory and imaging investigations, and indicators of unfavorable clinical outcomes.

The co-morbidity was also a significant factor in the time to recovery of children with bacterial meningitis. Hence, comparing children patients who haven’t comorbidity with children who have comorbidity, the expected time to recovery for children who haven’t comorbidity was increased by 8.7% as compared to children patients who have comorbidity, keeping the other variable factor remaining constant (ϕ = 1.098; 95% CI 1.022 1.164; P-value < 0.008). This shows that children who don’t have co-morbidity have a prolonged time to recovery as compared to children with co-morbidity. Comorbidity is linked to more complicated clinical management, poorer health outcomes, and higher medical expenses. Comorbidities might make treating a medical issue more difficult. The concept of burden of illness or disease, which is determined by the entire burden of physiological dysfunction or the total burden of all illness kinds that have an effect on a person's physiologic reserve, has also been expressed through the use of comorbidity. The examination of mechanisms such as direct causation, associated risk factors, heterogeneity, and independence that may contribute to the coexistence of many disorders in a patient is necessary, and the consequences for clinical care must be taken into account.

The breastfeeding of children also played a significant effect on the time to recovery of children with bacterial meningitis. Comparing the not-breathing children patient with breastfeeding children patient 1, the expected time to recovery for the no-breathing children patient was decreased by 48.4% as compared to the breastfeeding children bacteria patient, keeping the other covariates constant. (ϕ = 0.616; 95% CI 0.404–0.939; *p*-value < 0.024). This indicates that breastfeeding children have a greater chance of recovery than non-breathing children caused by bacterial meningitis disease. Human milk offers protection against distinct clinical ailments (such as necrotizing enter apathy, bacteremia, meningitis, respiratory tract infections, diarrheal diseases, and otitis media) as well as against certain pathogens (viruses, bacteria, and parasites). Breast milk is one of the most essential elements in preventing newborns from contracting infectious.

## Discussion

In this study, bacterial meningitis cases were common in 1 month to 1 year of age group (1–12 months) patients, which accounted for 45.42% of bacterial meningitis cases in pediatric departments. Other age groups, such as less than 1 month, between 13 and 36 months, 37–60 months, and greater than 60 months, accounted for 0.56, 20.37, 8.97, and 24.67%, respectively. This study was lower than the study in Swedish and Beverley [25, 26].

The age group of children had a significant effect on the time to recovery of children with bacterial meningitis. The median time for improvement was 5 days. The patients whose age is greater than 60 months had a significantly prolonged time of curing as compared to the patients aged less than 1 month. Numerous studies indicate that the following age groups are terminal: The most common age groups to be afflicted by bacterial neuro infections are infants and the elderly. Infants and newborns have a more permeable blood–brain barrier and a developing immune system [2]. Immune senescence, or the weakening of the immune system in individuals over 65, increases a person’s vulnerability to illnesses and reduces the effectiveness of vaccinations. In contrast, this observation was in line with the previous studies that had already shown that the younger age children patients have prolonged time to recover from the bacterial meningitis [1, 27].although, comparable to the research Children under the age of 12 months are the group most impacted by BM in France, Nigeria, Guatemala, and Malaysia1 [1]. According to Basri in Malaysia, BM increases the risk of death in children under the age of 1 year (OR = 3.13 [1.33–7.24] [5]. These findings are consistent with our research, which found that children with BM who are younger than 6 months old had a twice-higher chance of dying. Young newborns may need more specialized care because they are a population at risk for BM mortality [8].

Sex had a significant effect on the time to recovery of children with bacterial meningitis. The female patients had more time to recover as compared to the female patients, and, most similarly to the descriptive type of study that was carried out from May 2012 to April 2013, the male patients had 55.2% [28, 29]. According to reports from MacCormick in Malawi [30], Kuti in Nigeria [31], and Basri in Malaysia14, most BM impact studies revealed a 50–60% male preponderance. Each nation's sociodemographic composition affects the sex ratio. Sex, however, does not increase the risk of dying from BM.

The vaccinated group of patients had significantly shorter recovery times than the non-vaccinated group; in other words, the non-vaccinated group took longer to heal than the vaccinated group of patients who were successful in getting better. Similar to this, a prospective study on the cause of childhood acute bacterial meningitis was conducted, and this study had already demonstrated that the vaccinated group of patients, who made up 91.3% of the patient population, had better outcomes than the non-vaccinated group in terms of healing time [32]. Research from many nations’ shows that the PCV vaccination lowers the incidence of pneumonia that is mistakenly diagnosed as a respiratory virus infection as well as the usage of antibiotics. For instance, after PCV7 was made available in the United States in 2000, the number of antibiotic prescriptions for acute otitis media in children under the age of 2 years decreased by 42% between 1997 and 2004 [14]. An international panel of experts calculated that, across the 75 countries analyzed, universal PCV coverage could prevent up to 11.4 million days of antibiotic therapy for pneumonia caused by S. pneumonia each year in children under the age of five[18]. This represents a 47% reduction in antibiotic therapy.

The comorbidity of patients had a significant effect on the time to recovery of children with bacterial meningitis. Children who do not have co-morbidity have a prolonged time to recovery as compared to children with co-morbidity. This is also consistent with other research, such as a comparative cohort study that was conducted between 2014 and 2019, in which the co-morbidity of the patients was a significant factor in the patients' outcomes, and another study that was also conducted in Ethiopia, a retrospective chart [33, 34]. The presence of comorbidities was associated with the development of acute organ dysfunction (i.e., severe sepsis). Although the presence of comorbidities alters the choice of antibiotic in accordance with scientific societies' guidelines and is linked to an increased likelihood of treatment failure, these indications are primarily based on expert opinion rather than prospective studies [18]. Therefore, research is required to determine whether the comorbidities have any connection to the microbe that causes AE-COPD and whether this connection could aid in the empirical selection of an antibiotic. In this manner, the likelihood of resistance developing as well as treatment failure could be decreased [5].

The breastfeeding covariate had a significant effect on the time to recovery of children with bacterial meningitis. The breast feeding of the patient had a positive effect on the time of recovery, and this was consistent with the other study that was conducted in Rebro Country, Sweden, over a 6 year period, 1987–1992. Breastfeeding patients had a shorter curing time than those who were not breastfeeding while using the same covariate [35]. Also in the study in eastern Ethiopia, the acceleration factor showed that breastfeeding patients had a shorter curing time than those who were not breastfeeding while using the same covariate [29, 36].

## Conclusion

This study used the parametric shared frailty and AFT models to identify factors influencing the time to recovery of children with bacterial meningitis at Jigjiga University referral hospital in the Somali regional state of Ethiopia. The results of the parametric shared frailty and AFT models revealed that age group, comorbidity, sex, vaccination status, and breastfeeding were significant factors determining the time to recovery of children with bacterial meningitis at Jigjiga University referral hospital. Of these important variables, children with bacterial meningitis recovered more quickly when they were older and if they were non-comorbid. Conversely, there was a negative correlation found between children’s recovery time and sex, vaccinations, and breastfeeding. When compared to female child bacterial patients, the male child patients' anticipated recovery time was 7.9% shorter. Nevertheless, holding the other covariates constant, the number of children without comorbidity increased by 8.7% relative to the number of children with comorbidity, and the number of children without breathing decreased by 48.4% relative to the number of breastfeeding children of bacterial patients.

With esteem to log likelihood, AIC, and BIC values, the BM data set fit better. Consequently, the inverse Gaussian frailty model with the lognormal baseline was better than the lognormal Gamma frailty model, the log logistic Gamma frailty model, the Weibull-Gamma frailty model, the exponential-inverse Gaussian frailty model, and the exponential-Gamma frailty model. The variable of interest was significantly affected by clustering. The time to recovery of patients from the bacterial meningitis dataset is affected by clustering because of differences in the patients' time to recovery distribution among the 20 kebele from which they came.

## Recommendations

The study conclusions led to the following recommendations being made: Patients with a co-morbidity of bacterial meningitis should receive extra care from medical staff at the Jigjiga University referral hospital. Patients under 60 months of age must be made aware of the fact that their recovery times are longer than those of other age groups are. To get parents to vaccinate their children against bacterial meningitis, the nation's Ministry of Health and Jigjiga University Referral Hospital must inform the public about the seriousness of the disease. The public should become more aware of the illness and be open to the advice and viewpoints of medical experts.

## Limitation of study

The main limitation of the research was the utilization of secondary data, which may have omitted some important patient information such as house rowdiness the residents in a house rooms with two categories a- < 3individual per room and b- > 3individual per room, mother education (elementary, secondary schools and university), malnutrition (anemia), family income, pathogen (*L. monocytogenes and Nocardia spp.*), and high according to local master of living), children's weight, indicators, and symptoms.

## Data Availability

This article contains all of the information that was created or examined throughout the investigation.

## Abbreviations

AIC: Akaike information criteria
BM: Bacterial meningitis
CFR: Case fatality rate
CSF: Cerebrospinal fluid
CSM: Cerebrospinal meningitis
EC: Ethiopian calendar
IMD: Invasive meningococcal disease
JJU: Jigjiga University
Km: Kilometer
PCV: packed cell volume
SICU: Sophisticated intensive care units
SSA: Sub-Saharan Africa
WHO: World Health Organization

## References

[1] Scheld WM (2002). Pathophysiology of bacterial meningitis: mechanism (s) of neuronal injury. J Infect Dis.

[2] Dias S (2017). Sex-based differences in adults with community-acquired bacterial meningitis: a prospective cohort study. Clin Microbiol Infect.

[3] Yamamoto M (2007). Interferon-γ and tumor necrosis factor-α regulate amyloid-β plaque deposition and β-secretase expression in Swedish mutant APP transgenic mice. Am J Pathol.

[4] Pollard AJ, Perrett KP, Beverley PC (2009). Maintaining protection against invasive bacteria with protein–polysaccharide conjugate vaccines. Nat Rev Immunol.

[5] Gordon SB (2000). Bacterial meningitis in Malawian adults: pneumococcal disease is common, severe, and seasonal. Clin Infect Dis.

[6] Purwanto DS (2020). Isolation and identification of *Streptococcus* pneumoniae serotype 6B from a patient with bacterial meningitis infection in Jakarta, Indonesia. Access Microbiol.

[7] Dou Z-Z (2021). Clinical characteristics and outcome analysis of 94 children with brain abscess in Beijing: a single-center retrospective study. Pediatr Infect Dis J.

[8] Basri R (2015). Burden of bacterial meningitis: a retrospective review on laboratory parameters and factors associated with death in meningitis, Kelantan Malaysia. Nagoya J Med Sci.

[9] MacNeil JR (2021). Updated recommendations from the advisory committee on immunization practices for use of the Janssen (Johnson & Johnson) COVID-19 vaccine after reports of thrombosis with thrombocytopenia syndrome among vaccine recipients—United States, April 2021. Morb Mortal Wkly Rep.

[10] Jafri RZ (2013). Global epidemiology of invasive meningococcal disease. Popul Health Metrics.

[11] Chaudhuri A (2008). EFNS guideline on the management of community-acquired bacterial meningitis: report of an EFNS task force on acute bacterial meningitis in older children and adults. Eur J Neurol.

[12] Edmond K (2012). Long term sequelae from childhood pneumonia; systematic review and meta-analysis. PLoS ONE.

[13] Zhang X-X (2019). The diagnostic value of metagenomic next-generation sequencing for identifying *Streptococcus* pneumoniae in paediatric bacterial meningitis. BMC Infect Dis.

[14] Sadeq H (2017). Childhood meningitis in Kuwait in the era of post pneumococcal conjugate vaccination: a multicenter study. J Infect Public Health.

[15] Organization WH (2013). Pocket book of hospital care for children: guidelines for the management of common childhood illnesses.

[16] Christiaensen L, Alderman H (2004). Child malnutrition in Ethiopia: can maternal knowledge augment the role of income?. Econ Dev Cult Change.

[17] Salih MMA (1991). Long term sequelae of childhood acute bacterial meningitis in a developing country: a study from the Sudan. Scand J Infect Dis.

[18] Adriani K (2012). Bacterial meningitis in pregnancy: report of six cases and review of the literature. Clin Microbiol Infect.

[19] Rocha AJD (2016). Modern techniques of magnetic resonance in the evaluation of primary central nervous system lymphoma: contributions to the diagnosis and differential diagnosis. Revista Brasileira de Hematologia e Hemoterapia.

[20] Di Sabatino A (2005). Depletion of immunoglobulin M memory B cells is associated with splenic hypofunction in inflammatory bowel disease. Off J Am College Gastroenterol.

[21] Keiding N, Andersen PK, Klein JP (1997). The role of frailty models and accelerated failure time models in describing heterogeneity due to omitted covariates. Stat Med.

[22] Mulugeta G, Tesfaye D, Tegegne AS (2022). Predictors for the duration of breastfeeding among ethiopia women of childbearing age with babies; application of accelerate failure time and parametric shared frailty models. BMC nutrition.

[23] de Jonge RC (2010). Predicting sequelae and death after bacterial meningitis in childhood: a systematic review of prognostic studies. BMC Infect Dis.

[24] Economou P, Caroni C (2005). Graphical tests for the assumption of gamma and inverse Gaussian frailty distributions. Lifetime Data Anal.

[25] Alemu M (2019). Determinants of neonatal sepsis among neonates in the northwest part of Ethiopia: case-control study. Ital J Pediatr.

[26] Indermun S (2014). Current advances in the fabrication of microneedles for transdermal delivery. J Control Release.

[27] Dätwyler, C. and T. Stucki, Parametric survival models. 2011.

[28] Golding N (2017). Mapping under-5 and neonatal mortality in Africa, 2000–15: a baseline analysis for the sustainable development goals. Lancet.

[29] Maes HH, Neale MC, Eaves LJ (1997). Genetic and environmental factors in relative body weight and human adiposity. Behav Genet.

[30] McCormick DW (2013). Risk factors for death and severe sequelae in Malawian children with bacterial meningitis, 1997–2010. Pediatr Infect Dis J.

[31] Kuti BP (2015). Epidemiological, clinical and prognostic profile of childhood acute bacterial meningitis in a resource poor setting. J Neurosci Rural Pract.

[32] Acevedo R (2019). The Global Meningococcal Initiative meeting on prevention of meningococcal disease worldwide: epidemiology, surveillance, hypervirulent strains, antibiotic resistance and high-risk populations. Expert Rev Vaccines.

[33] Xiang Y-T (2020). Timely mental health care for the 2019 novel coronavirus outbreak is urgently needed. Lancet Psychiatry.

[34] Tilahun T, Sinaga M (2016). Knowledge of obstetric danger signs and birth preparedness practices among pregnant women in rural communities of eastern Ethiopia. Int J Nurs Midwifery.

[35] Stewart EJ (2012). Growing unculturable bacteria. J Bacteriol.

[36] Erdem I (2008). Clinical features, laboratory data, management and the risk factors that affect the mortality in patients with postoperative meningitis. Neurol India.

